# A Study on the Sound Insulation Performance of Cross-laminated Timber

**DOI:** 10.3390/ma14154144

**Published:** 2021-07-25

**Authors:** Jui-Yen Lin, Chieh-Ting Yang, Yaw-Shyan Tsay

**Affiliations:** Department of Architecture, National Cheng Kung University, Tainan 70101, Taiwan; xryan19891207@gmail.com (J.-Y.L.); iochiyomi@gmail.com (C.-T.Y.)

**Keywords:** transmission loss, predictive models, wooden structure

## Abstract

Cross-laminated Timber (CLT) has become an emerging board material of wood construction that is strong enough to sustain a high-rise building. However, many wooden congregate housing units overseas that utilize CLT have poor sound environments because the low mass of such wood influences sound insulation performance. In this research, we explored the effect of different CLT walls on sound insulation performance and integrated applicable sound insulation simulation tools to simplify the process of designing a CLT wall structure. This research aimed at a double wall and CLT combined with a gypsum board as the research object. The sound insulation performance test was carried out in a laboratory, while the sound insulation performance of the structure was predicted through simulation tools and prediction models and then compared with the measured values to verify the applicability of the simulation tool. The CLT with a double wall and CLT with gypsum board (CLT + GB) achieved $R_{w}$ of 50 dB. The numerical simulation had better prediction performance than INSUL at the double wall, while the double wall with cavity structure was close to the measured result via mass law calculation. The INSUL-predicted CLT with a gypsum board at 500 Hz~3150 Hz was close to the measured value.

## 1. Introduction

In recent years, the growth of cross-laminated timber (CLT) has been widely researched with regard to the special characteristics of this material, mainly concerning its economic and environmental benefits, carbon reduction, building design, and energy efficiency. While energy reduction has been the main direction [1], some aspects still have not been fully analyzed, including the noise protection that CLT walls can provide to the possible application of building elements.

CLT was first developed in Europe in the mid-1990s [2]. CLT is an orthogonal arrangement of layers of dimension lumber boards adhesive together to make a more stable and durable structure than previous wood panels and can be directly used on floors, roofs, walls, and other parts. It can serve as an alternative material to concrete and steel [3,4,5,6,7]. The specific gravity of CLT is about 1/6 of that of reinforced concrete. It is prefabricated in a factory and then assembled on-site, which can considerably shorten the construction time [8,9,10]. The CLT construction method has gradually developed and matured, and architects have begun to propose the possibility of various high-rise wooden structures. Considering the substantive technical aspect, Van De Kuilen et al. [11] proposed a hybrid structure with a concrete core tube and CLT, potentially offering CLT more diverse applications.

With regard to the building acoustic environment of living, the International Building Code (IBC) regulates a transmission loss class of no less than 50 on walls, partitions, and floor-ceiling [12]. The characteristics of low-density CLT are advantageous for the strength of seismic structures but are disadvantageous for sound insulation performance. Many cases of CLT wooden structure assembled houses were evaluated after use, and it was found that the sound environment performance was not sufficient which is comparing the common material of the wall, such as concrete [13,14,15,16]. In general, the 15 cm to 18 cm of concrete wall performance of the sound insulation achieved 50 dB. To improve the quality of the indoor acoustic environment of wooden buildings, we first need to grasp the current situation of the sound insulation performance of CLT structures to facilitate subsequent research on improvement methods.

In the past, studies conducted single-board or multi-layer board experiments or numerical simulations on the sound insulation performance of CLT [17,18], and the results show that the CLT panel with a thickness of 100 mm has a sound insulation performance of 34 dB. In addition, the 100 mm CLT with 2.5 mm and 12.5 mm plasterboard have a sound insulation performance of 45 dB. Therefore, CLT of different tree species and thicknesses has different performances in sound insulation performance. CLT has a wide variety of tree species and different densities. Common CLT is composed of Douglas fir, Western hemlock, spruce, and other coniferous trees, but different tree species had different sound insulation performances. Most of the tree species included in the recent research use spruce, and there is less information on the CLT sound insulation performance of other tree species such as cypress. In addition, these studies all use experimental methods, and less research discusses the prediction methods of CLT sound insulation performance or comparison prediction tools.

In order to reduce the cost of development, it is necessary to conduct research and development through simulation tools. However, few studies have compared simulation tools with measured values regarding sound insulation performance, and few studies compare the prediction results of several simulation tools at the same time.

This research into the sound insulation performance of CLT walls explores the difference between transmission loss of the single wall, double wall, and CLT + GB. We studied the effect of different construction methods on sound insulation performance and proposed a structural form that met sound insulation regulations, thus determining an applicable simulation tool that can become a reference for the sound insulation structure in the subsequent development of CLT construction projects.

## 2. Materials and Methods

### 2.1. Specimen Boundary Condition

**Figure 1:**
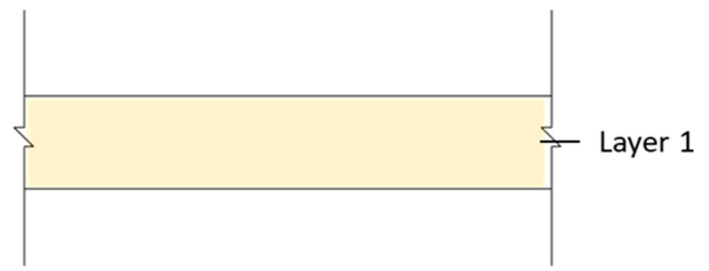
The diagram of the single wall (A1).

**Figure 2:**
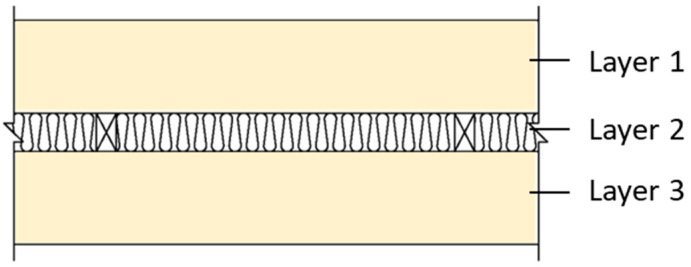
The diagram of the double wall (B1–B8).

**Figure 3:**
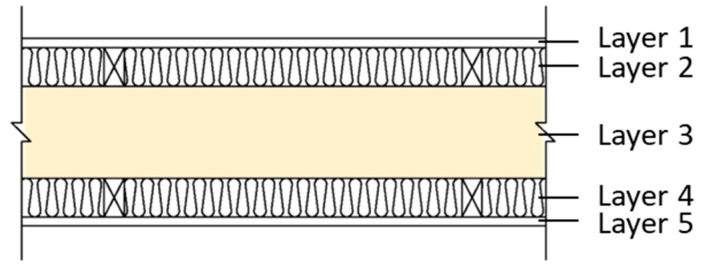
The diagram of CLT + GB (C1–C4).

**Figure 4:**
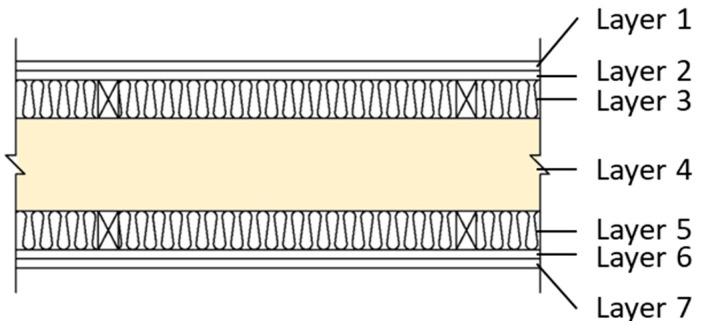
The diagram of CLT + GB (C5).

In this study, we selected a CLT made from hinoki cypress, which is commercially produced as the test specimen. The material specifications are shown in Table 1. Due to the limitation of CLT manufacturing technology and specifications, this research used a 750 mm x 1500 mm specimen, which is adhesive by 3 ply (2 longitudinal layer, 1 transverse layer), 120 mm thick, and each ply is 40 mm thick. The sound insulation performance of the CLT walls with different structures analyzed in this research was divided into three different wall structures: which is single wall, double wall, and CLT with gypsum board (CLT + GB), as shown in Figure 1, Figure 2, Figure 3 and Figure 4. Then, we tested 14 specimens, as shown in Table 2.

The assembly method of the test specimen made by the glass wool was filled in a wooden frame then screw with the gypsum board. Meanwhile, the super-stick tape was attached to the CLT and wooden frame with a gypsum board.

### 2.2. Laboratory Environment

**Figure 5:**
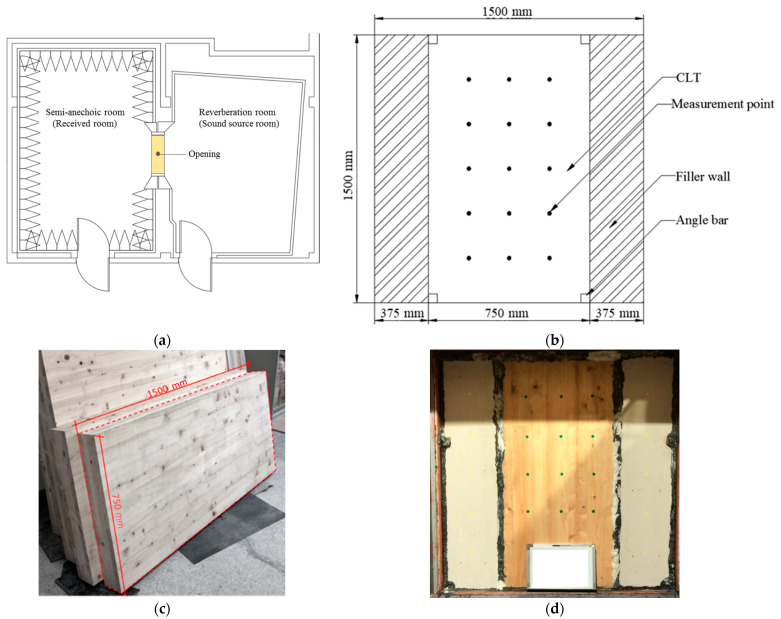
The installation of the test area and implementation. (**a**)The specimen was implemented in the opening area, and the measurement area was 1.125 m^2^. (**b**) Evenly distributed measurement points. (**c**) The sample of CLT panel (dashed range). (**d**) The CLT was installed in the middle of the area.

Our research was divided into laboratory measurement and sound insulation simulation prediction analysis. The laboratory measurement focused on the sound insulation effect of wall panels and construction materials and then valued the sound insulation index in accordance with ISO 717-1 [19] and ASTM E413 [20]. The evaluation value obtained by ISO is the weighted sound reduction index ($R_{w}$), and ASTM is the sound transmission class (STC) which are the common application to evaluate the performance of materials. Measurements were then carried out according to the sound intensity method of ISO 15186-1 [21] and ASTM E2249 [22]. Due to this research using sound intensity measurement [21,22]. Therefore, the facility was built with two different rooms, which are a reverberation room and a semi-anechoic room. The reverberation room is a sound source room that generates sound energy; meanwhile, a semi-anechoic room is full of sound-absorbing wedges unless floor, which is for a room of received the sound. Furthermore, the instrument of measure sound intensity, including the B&K 2250 sound level meter/analyzer, omnidirectional sound source, sound intensity microphone, etc., was used in this research. The volume of the semi-anechoic room was 211.6 m^3^, and that of the reverberation room was 171.3 m^3^, as shown in Figure 5.

### 2.3. Prediction Approaches

In this research, we applied three models and one simulation tool to predict the transmission loss of different structures, and they were mass law, Sharp (1978) [23], Sasao (2006) [24,25,26], and INSUL [27]. Mass law is given by Equation (1). The density of CLT time the thickness of CLT, which is the CLT panel mass value and calculated substituted into Equations (1)–(7). Moreover, The value of TL could be used to calculate the $R_{w}$, which according to ISO 717, is the sum of unfavorable deviation requirements (not more than 32.0 dB).

(1) TL=20log(mf)−48

where *m* is the surface mass (kg/m^2^), and *f* is the frequency (Hz).

Sharp (1978) developed a series of models to predict the transmission loss of cavity walls, including without-connection walls, point-connection walls, and line-connection walls. In this paper, we presented the line-connection model, which is given by Equations (2)–(7).

(2) $$TL=\{\begin{array}{c} TL_{M},\;f<f_{0}\\ \;TL_{m1}+TL_{m2}+20\log\left(f_{0}d\right)-29,\;f_{0}<f<f_{b}\\ TL_{M}+\Delta TL_{M},\;f>f_{b} \end{array}$$

(3) $$TL_{M}=20\log(mf)-48$$

(4) $$f_{0}=113/\sqrt{m_{e}d}$$

(5) $$m_{e}=2m_{1}m_{2}/\left(m_{1}+m_{2}\right)$$

(6) $$\Delta TL_{M}=10\log\left(bf_{c1}\right)+K-18$$

(7) $$K=20\log\left[m_{1}/\left(m_{1}+m_{2}\right)\right]$$

where $TL_{M}$, $TL_{m1}$, and $TL_{m2}$ are the transmission loss values (dB) calculated from Equation (3); m is the surface mass (kg/m^2^) for the total construction (M = m_1_+m_2_); $f_{0}$ is the fundamental frequency (Hz) of mass-air-mass resonance and given by Equation (4); $f_{b}$ is the bridging frequency (Hz) at which the sound bridges begin to determine transmission loss; d is the width of the air cavity (m) for the line-connection cavity wall; $\Delta TL_{M}$ is given by Equation (6), where b is the spacing of line connections (m), and K is given by Equation (7).

Sasao (2006) uses the relationship between the fundamental sound characteristics and sound insulation characteristics of the wall to obtain a simulation prediction tool via excel VBA using the transfer matrix.

## 3. Results and Discussion

### 3.1. Sound Insulation Performance of CLT

#### 3.1.1. Single Wall of CLT

Herein, confirmative wood or timber material is considered a disadvantage compared with other building elements regarding sound insulation performance. Previous research has pointed out that in the sound insulation performance of single-board CLT, $R_{w}$ is 34 dB of 100 mm CLT [17]. When Schoenwald et al. [18] proposed a thickness with 78 mm and 175 mm of CLT, the sound insulation index $R_{w}$ was 33 dB and 41 dB, respectively.

**Figure 6:**
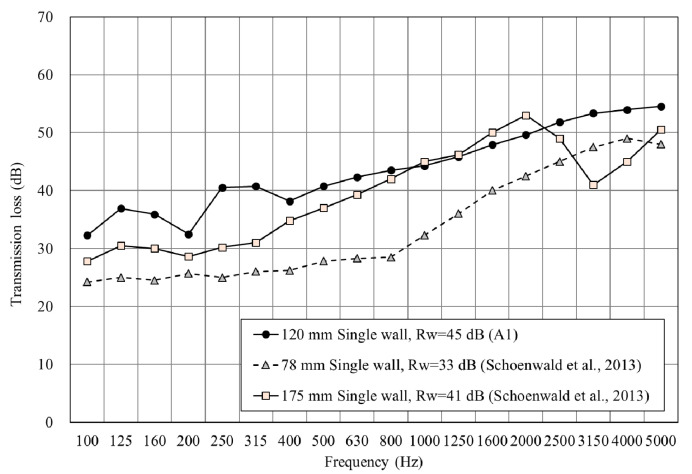
The sound insulation performance of the single wall.

As shown in Figure 6, the single wall (A1) of CLT achieved $R_{w}$ of 45 dB without other compounded materials in this study. Compared with other research, this finding shows a great foundation of sound insulation performance and is conducive to the subsequent design of various wall structures that can more effectively improve sound insulation performance. Furthermore, the sound insulation performance of a single wall achieved the requirement of adjacent spaces on private spaces in Leadership in Energy and Environmental Design (LEED).

#### 3.1.2. Double Wall of CLT

**Figure 7:**
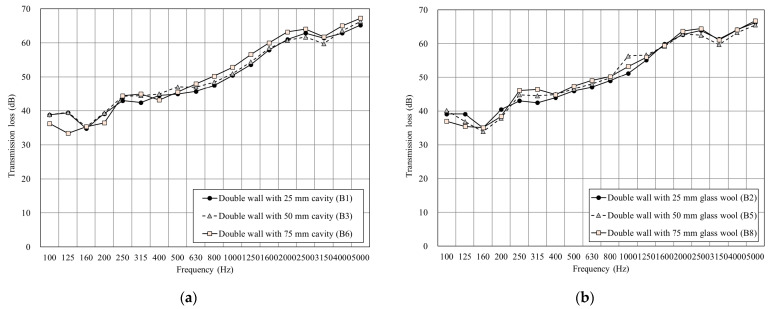

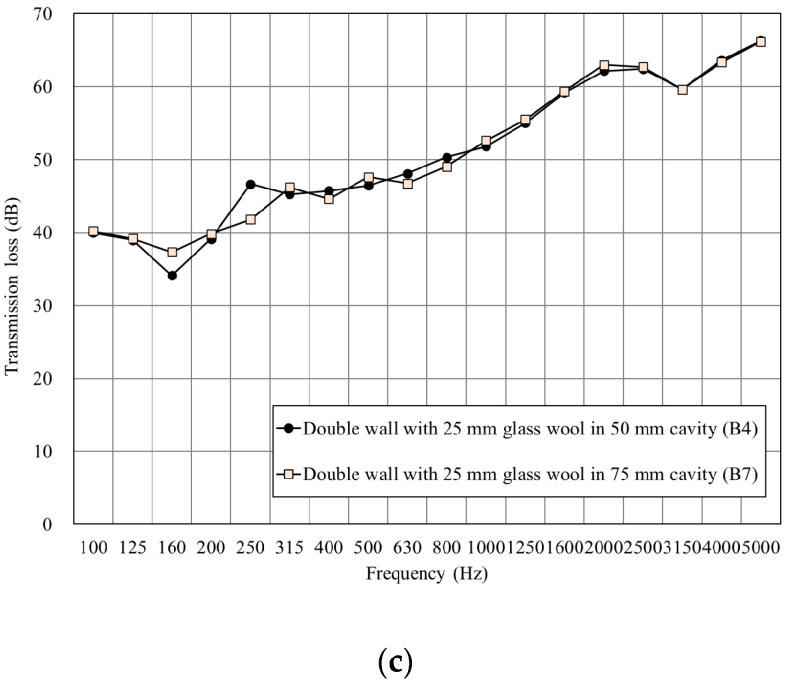
The sound insulation performance of the double wall: (**a**) Comparison of the test specimens with different cavity thicknesses. (**b**) Comparison of the test specimens with different glass wool thicknesses. (**c**) Comparison of the test specimens with different cavity thicknesses but the same thickness of glass wool.

As shown in Figure 7a, increasing the thickness of the cavity from 25 mm to 75 mm can improve the sound insulation on medium and high frequencies by 1 dB–3 dB. Furthermore, the double-wall resonance that occurred at 125 Hz and 160 Hz influenced the sound insulation performance. Figure 7b,c shows that the double wall was filled with glass wool in the cavity, which made few differences in the overall sound insulation performance, only increasing by 2 dB–3 dB at 500 Hz. This result shows that the small surface density of glass wool and a thin cavity cannot provide higher sound insulation performance, while the trend of transmission loss was similar.

#### 3.1.3. CLT + GB

**Figure 8:**
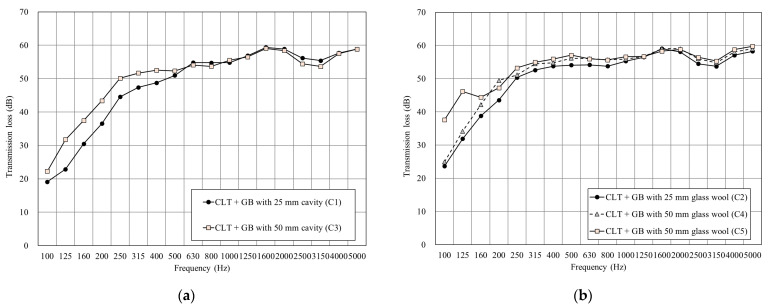
The sound insulation performance of CLT + GB. (**a**) Comparison of the test specimens with different cavity thicknesses. (**b**) Comparison of the test specimens with different glass wool thicknesses.

Figure 8 shows the results of CLT + GB with a different cavity and glass wool thicknesses. Figure 8a shows that increasing the thickness of the cavity increased the transmission loss under 500 Hz. However, the density of air is smaller than glass wool, so the sound insulation performance is not significantly improved at a high frequency. Figure 8b shows that filling the cavity with 48 K glass wool significantly improved the transmission loss under 500 Hz, while the linearity of the mid and high frequencies was similar. Furthermore, adding a piece of gypsum board on each side of the surface was able to greatly improve the overall sound insulation performance, especially at 100 Hz–125 Hz.

Table 3 shows that all the double walls of CLT achieved 50 dB on sound insulation performance. Furthermore, the surface mass of C1 was smaller than all the double-wall sets and resulted in poor sound insulation performance. The double wall in this research also achieved the acoustical requirements of IBC [14] on wall regulations.

### 3.2. Predict and Analysis of Sound Insulation

In order to reduce the cost of the subsequent development of CLT wall structures, we aimed to propose a feasible structure through simulation tools in the early stage and then conduct experiments to confirm said structure’s performance. Therefore, it was first necessary to verify whether the predictive ability of the simulation tool had a certain degree of reproducibility.

According to the sound insulation simulation model formula, the main parameter that affects sound insulation performance is material characteristics. The thickness, density, Young’s modulus, and Poisson’s ratio of the material were all necessary information. Furthermore, we needed to know the thickness, density, and flow resistance of the plate of the filling materials, as shown in Table 1.

This study used three theoretical prediction models and INSUL [25] for discussion and analysis. We integrated the applicability of the predicted models via the difference in sound insulation performance between experimental and predicted results.

#### 3.2.1. Sound Insulation Simulation and Analysis of a Double Wall

First, we had to confirm the double-plate structure of resonance frequency and the critical frequency, which predict the location where the low-frequency sound insulation value drops, the double-plate resonance frequency (Equation (8)), and the critical frequency (Equation (9)). The resonance frequency of the double wall was 51 Hz, lower than the measurement range. Meanwhile, the critical frequency calculated at 141 Hz was close to the experimental result, which occurred at 160 Hz.

(8) $$f_{r}=\frac{1}{2\pi }\sqrt{\;\frac{\rho c^{2}}{d}\left(\frac{1}{m_{1}}+\frac{1}{m_{2}}\right)}$$

(9) $$f_{c}=\frac{c^{2}}{2\pi }\sqrt{\;\frac{12m\left(1-\sigma^{2}\right)}{Eh^{3}}}$$

where $f_{r}$ is the double resonant frequency (Hz), ρ is the air density (kg/m^3^), m is the surface density of double panel, c is the speed of sound in air (m/s), d is the distance between the two panels (m), $f_{c}$ is the critical frequency, σ is Poisson’s ratio, E is Young’s modulus (Pa), and h is the thickness (m).

**Figure 9:**
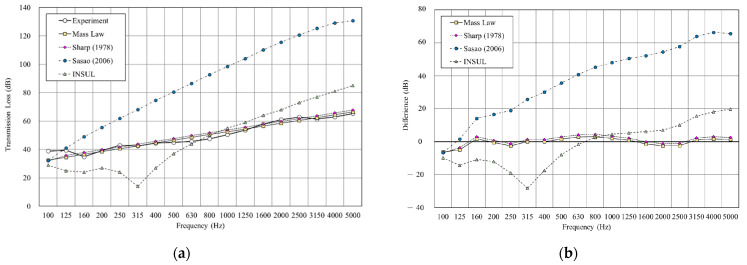
Comparison between experimental and predicted results of the double wall with 25 mm cavity (B1). (**a**) The sound insulation performance of experiment and prediction. (**b**) The RMSE results of different prediction models.

As shown in Figure 9, we used the sound insulation prediction and simulation tool to simulate a double-layer structure with an air layer of 25 mm. The result shows that the root-mean-square error (RMSE) of mass law is 2.6 dB, Sharp’s model is 3.0, at a minimum. The RMSE of INSUL and Sasao’s models showed large differences from the experimental value. However, INSUL is the only prediction tool that considers the resonance frequency, although the predicted results deviate from the measured value.

**Figure 10:**
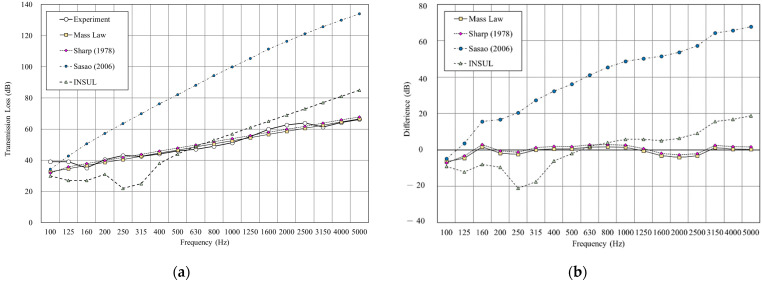
Comparison between experimental and predicted results of the double wall with 25 mm glass wool (B2). (**a**) The sound insulation performance of experiment and prediction. (**b**) The RMSE results of different prediction models.

Figure 10 shows the double wall with glass wool filled in a 25 mm cavity. The result shows that both mass law and Sharp’s prediction were close to the experimental result, with the RMSE of mass law 2.6 dB and Sharp’s model 2.7 dB. However, the INSUL and Sasao’s models deviated still considerably from the measured value. Although the prediction result of INSUL is slightly improved by filling glass wool, it is still far from the measured value.

**Figure 11:**
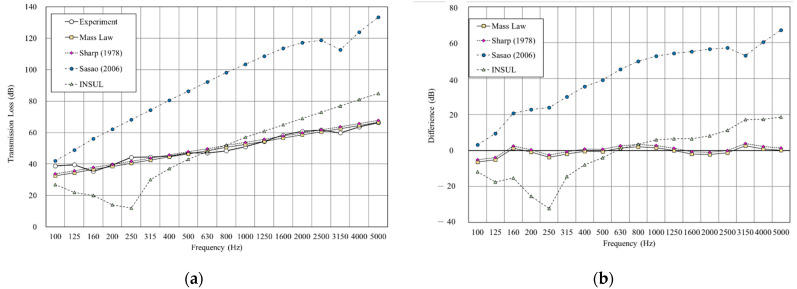
Comparison between experimental and predicted results of the double wall with 50 mm cavity (B3). (**a**) The sound insulation performance of experiment and prediction. (**b**) The RMSE results of different prediction models.

**Figure 12:**
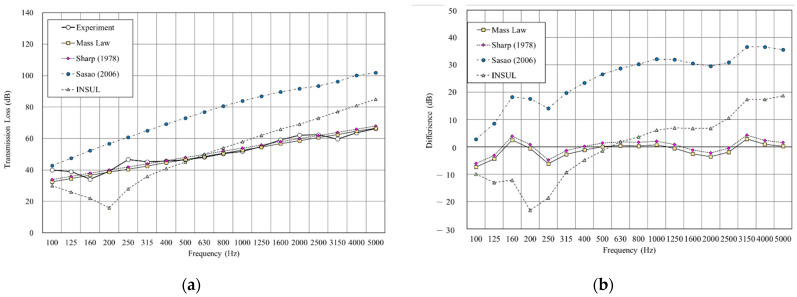
Comparison between experimental and predicted results of the double wall with 25 mm glass wool in 50 mm cavity (B4). (**a**) The sound insulation performance of experiment and prediction. (**b**) The RMSE results of different prediction models.

**Figure 13:**
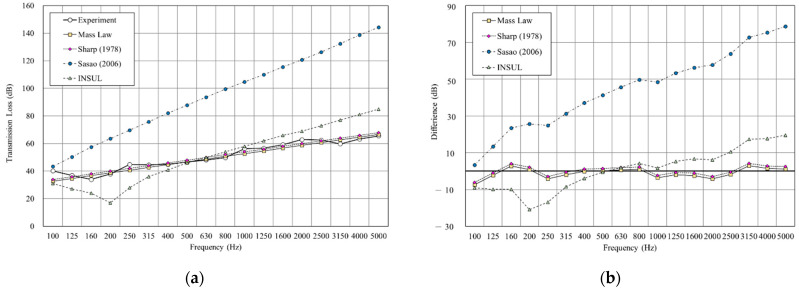
Comparison between experimental and predicted results of the double wall with 50 mm glass wool (B5). (**a**) The sound insulation performance of experiment and prediction. (**b**) The RMSE results of different prediction models.

**Figure 14:**
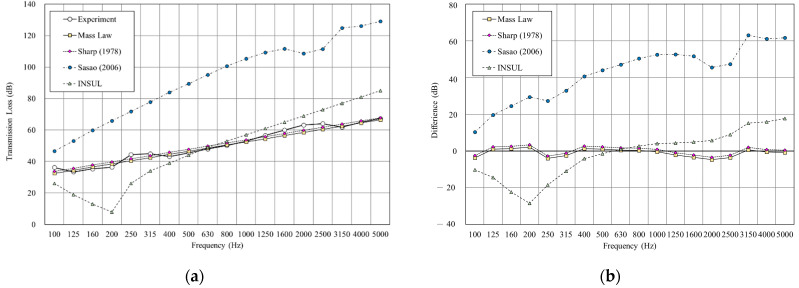
Comparison between experimental and predicted results of the double wall CLT with 75 mm cavity (B6). (**a**) The sound insulation performance of experiment and prediction. (**b**) The RMSE results of different prediction models.

**Figure 15:**
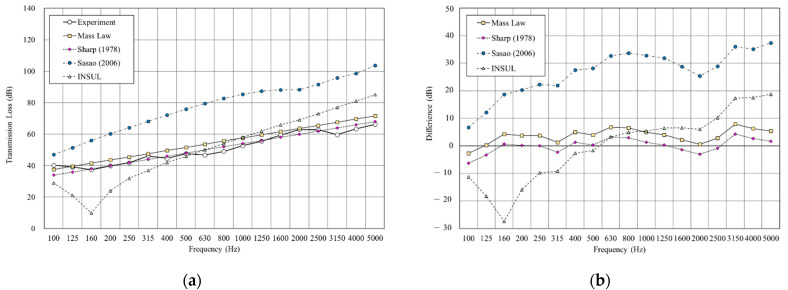
Comparison between experimental and predicted results of the double wall with 25 mm glass wool in 75 mm cavity (B7). (**a**) The sound insulation performance of experiment and prediction. (**b**) The RMSE results of different prediction models.

**Figure 16:**
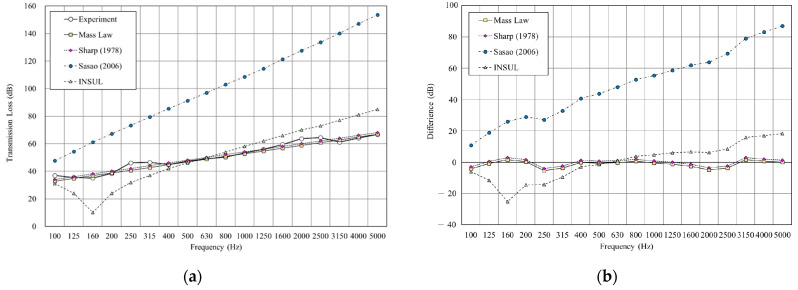
Comparison between experimental and predicted results of the double wall with 75 mm glass wool (B8). (**a**) The sound insulation performance of experiment and prediction. (**b**) The RMSE results of different prediction models.

Figure 11, Figure 12, Figure 13,Figure 14, Figure 15 and Figure 16 show the prediction results of the sound insulation performance of a double wall with different glass wool thicknesses in 50 mm cavities. Overall, mass law and the Sharp (1978) model were the closest to the measured values, while Sasao (2006) and INSUL deviated from the experimental values. Figure 9, Figure 11 and Figure 14 show that on the double-wall with an air cavity, Sasao (2006) had resonance at high-frequency that was the same as the experimental value.

As described above, mass law and the Sharp (1978) model are suitable for predicting double walls of CLT, while the prediction results of Sasao (2006) and INSUL have insignificant prediction results; however, they were the only predictions that occurred in resonance.

#### 3.2.2. Sound Insulation Simulation and Analysis of CLT + GB

A sandwich form is commonly used in buildings for their wall system. In this study, we used gypsum board (thickness: 12 mm) and filling materials (glass wool) as the materials, which were covered by CLT as the main structure. We then conducted the measurement and simulation analysis of the sound insulation performance of different thicknesses.

**Figure 17:**
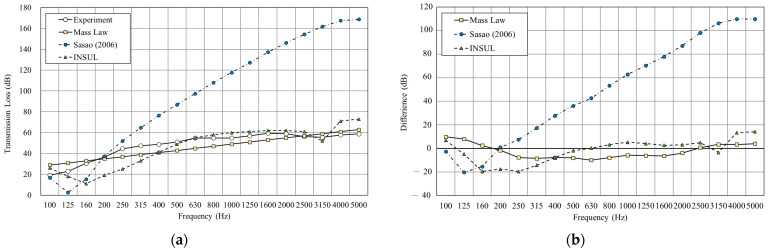
Comparison between experimental and predicted results of CLT + GB with 25 mm cavity (C1). (**a**) The sound insulation performance of experiment and prediction. (**b**) The RMSE results of different prediction models.

**Figure 18:**
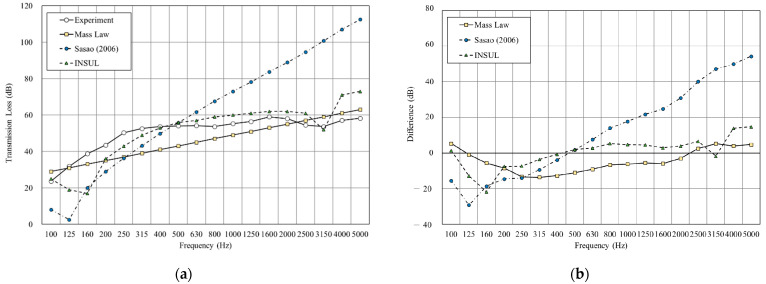
Comparison between experimental and predicted results of CLT + GB with 25 mm glass wool (C2). (**a**) The sound insulation performance of experiment and prediction. (**b**) The RMSE results of different prediction models.

**Figure 19:**
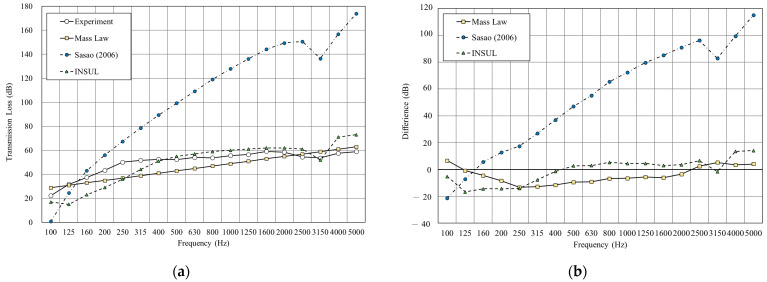
Comparison between experimental and predicted results of CLT + GB with 50 mm cavity (C3). (**a**) The sound insulation performance of experiment and prediction. (**b**) The RMSE results of different prediction models.

**Figure 20:**
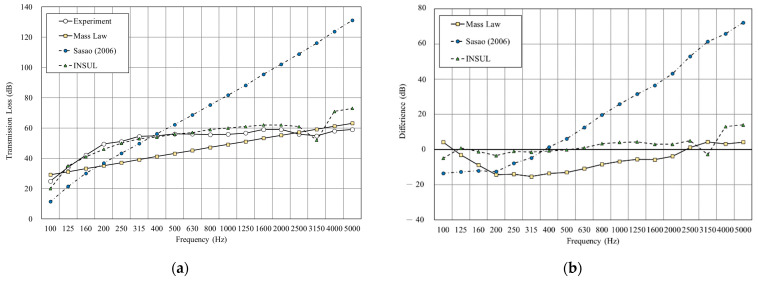
Comparison between experimental and predicted results of CLT + GB with 50 mm glass wool (C4). (**a**) The sound insulation performance of experiment and prediction. (**b**) The RMSE results of different prediction models.

Figure 17, Figure 18, Figure 19 and Figure 20 show the prediction results of CLT + GB filled with glass wool in the different cavities. As the basic theory of mass law is an ideal equation, a stable oblique straight line will occur in predictions, which is not conducive to predicting complex wall structures, although RMSE is the smallest of all predictions. The Sasao (2006) structure for the overall prediction of CLT + GB still deviates from the measured value. INSUL was the only result that had the same linear trend as the measured value and compared with the others, the RMSE of 500 Hz–3150 Hz was closer to the measured value.

**Figure 21:**
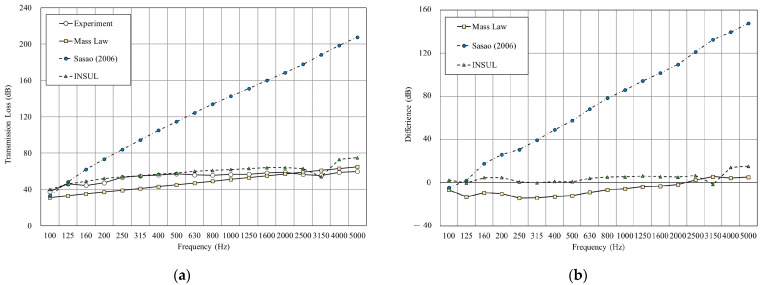
Comparison between experimental and predicted results of CLT + GB with 50 mm glass wool and twice the Gypsum board than in C4 (C5). (**a**) The sound insulation performance of experiment and prediction. (**b**) The RMSE results of different prediction models.

As shown in Figure 21, INSUL achieved better prediction results when simulating more complex wall structures. These results show the transmission loss difference between 0 and 1.1 at 250 Hz and 500 Hz; furthermore, the overall linear trend was similar to the measured value. The resonance at 3150 Hz was also the same as the measured value.

As shown in Table 4, the predictive simulation of mass law is more suitable for the double wall than the other predictive simulation tools. Sasao (2006) is not applicable for predicting the double wall and CLT + GB. INSUL is the only tool that can predict the three obvious slope intervals similar to the actual measurement results, and the resonance frequency appears at 3150 Hz, the same as the actual measurement.

## 4. Conclusions

The results of the basic performance analysis of the single-wall CLT sound insulation show that the $R_{w}$ and STC are 45 dB. However, the above results only meet the required sound insulation performance. It was not enough for other requirements of walls in different spaces. Therefore, this study proposes a double-layer structure and a CLT + GB structure for sound insulation performance analysis.

In this study, the double wall and CLT + GB demonstrated a sound insulation performance of $R_{w}$ greater than 50, except for C1. Filling with glass wool or changing the thickness of the cavity within the range of 50 mm, had less influence on the overall performance, and the improvement effect was below 2 dB. The transmission loss of CLT + GB had advantages in all frequency bands, which can increase the overall performance by 3 dB.

Both mass law and Sharp (1978) can be applied to predict the sound insulation performance of the double wall. Compared with all the prediction methods, the RMSE values of mass law and Sharp (1978) were smaller, 2.3 dB–3.0 dB and 2.2 dB–3.0 dB, respectively. Therefore, considering the applicability, simplicity, and efficiency, we recommend using mass law first when predicting the sound insulation performance for double-wall construction. For RMSE, the value is lower and better. To unify the double-wall results, it shows thst the R square result of mass law and Sharp (1978) was 0.93–0.96.

The results showed that when the structure was thicker, and the cavity was filled with glass wool, INSUL prediction results were better. Furthermore, INSUL can significantly simulate a linear trend similar to the measured value in predicting multi-layer wall structures.

In summary, the result shows the sound insulation performance of CLT is greatly affected by mass. Therefore, CLT made from other denser tree species may have better sound insulation performance. As predicted, Sharp (1978) and mass law could be chosen for the double wall. INSUL could be selected to simulate the multi-layer wall structures.

## Figures and Tables

**Table 1:** Boundary conditions of wall materials.

Material	Thickness (mm)	Size(mm)	Density(kg/m^3^)	Young’s Modulus (GPa)	Poisson Ratio	Friction Loss (N·s/m^4^)
CLT	120	750 × 1500	441	5.25	0.4	-
Gypsum	12		710	2	0.4	-
Glass wool	25/50/75		48	-	-	27,000

**Table 2:** Test specimen settings.

Type	No.	Materials (Thickness, mm)							Total Thickness (mm)
		Layer 1	Layer 2	Layer 3	Layer 4	Layer 5	Layer 6	Layer 7	
Single wall	A1	CLT (120)	-	-	-	-	-	-	120
Double wall	B1	CLT (120)	Cavity (25)	CLT (120)	-	-	-	-	265
	B2		Glass wool (25)						
	B3	CLT (120)	Cavity (50)	CLT (120)	-	-	-	-	290
	B4		Glass wool (25) in cavity (50)						
	B5		Glass wool (50)						
	B6	CLT (120)	Cavity (75)	CLT (120)	-	-	-	-	315
	B7		Glass wool (25) in cavity (75)						
	B8		Glass wool (75)						
CLT + GB	C1	Gypsum (12)	Cavity (25)	CLT (120)	Cavity (25)	Gypsum (12)	-	-	194
	C2		Glass wool (25)		Glass wool (25)				
	C3	Gypsum (12)	Cavity (50)	CLT (120)	Cavity (50)	Gypsum (12)			244
	C4		Glass wool (50)		Glass wool (50)				
	C5	Gypsum (12)	Gypsum (12)	Glass wool (50)	CLT (120)	Glass wool (50)	Gypsum (12)	Gypsum (12)	268

**Table 3:** The results of sound insulation performance.

Type	Single Wall	Double Wall								CLT + GB				
No.	A1	B1	B2	B3	B4	B5	B6	B7	B8	C1	C2	C3	C4	C5
Total thickness (mm)	120	265		290			315			194		244		268
$R_{w}$ (dB)	45	50	51	50	50	50	51	50	52	49	53	53	55	57
STC	45	50	51	50	50	50	51	50	52	46	54	54	56	57

**Table 4:** Analysis of applicable methods with the different prediction tools for the CLT wall structure.

Prediction Tool	Double Layer Structure	CLT + GB
Mass law	Applicable	1. Not applicable under 2000 Hz 2. Applicable for 2000 Hz~500 Hz
Sharp (1978)	Applicable	-
Sasao (2006)	Not applicable	Not applicable
INSUL	Not applicable	Applicable

## Data Availability

The data presented in this study are available upon request from the corresponding author.
